# Effect of Varying Hemodynamic and Vascular Conditions on Fractional Flow Reserve: An In Vitro Study

**DOI:** 10.1161/JAHA.116.003634

**Published:** 2016-06-30

**Authors:** Kranthi K. Kolli, James K. Min, Seongmin Ha, Hilary Soohoo, Guanglei Xiong

**Affiliations:** ^1^ Dalio Institute of Cardiovascular ImagingWeill Cornell Medical CollegeNew YorkNY; ^2^ Departments of Radiology and MedicineWeill Cornell Medical CollegeNew YorkNY

**Keywords:** 3D printing, aortic pressure, coronary artery disease, fluid dynamics, fractional flow reserve, in vitro, myocardial infarction, non–myocardial infarction, Basic Science Research, Coronary Circulation, Blood Pressure, Ischemia, Catheter-Based Coronary and Valvular Interventions

## Abstract

**Background:**

The aim of this study was to investigate the impact of varying hemodynamic conditions on fractional flow reserve (ratio of pressure distal [P_d_] and proximal [P_a_] to stenosis under hyperemia) in an in vitro setting. Failure to achieve maximal hyperemia and the choice of hyperemic agents may have differential effects on coronary hemodynamics and, consequently, on the determination of fractional flow reserve.

**Methods and Results:**

An in vitro flow system was developed to experimentally model the physiological coronary circulation as flow‐dependent stenosis resistance in series with variable downstream resistance. Five idealized models with 30% to 70% diameter stenosis severity were fabricated using VeroClear rigid material in an Objet260 Connex printer. Mean aortic pressure was maintained at 7 levels (60–140 mm Hg) from hypotension to hypertension using a needle valve that mimicked adjustable microcirculatory resistance. A range of physiological flow rates was applied by a steady flow pump and titrated by a flow sensor. The pressure drop and the pressure ratio (P_d_/P_a_) were assessed for the 7 levels of aortic pressure and differing flow rates. The in vitro experimental data were coupled with pressure–flow relationships from clinical data for populations with and without myocardial infarction, respectively, to evaluate fractional flow reserve. The curve for pressure ratio and flow rate demonstrated a quadratic relationship with a decreasing slope. The absolute decrease in fractional flow reserve in the group without myocardial infarction (with myocardial infarction) was on the order of 0.03 (0.02), 0.05 (0.02), 0.07 (0.05), 0.17 (0.13) and 0.20 (0.24), respectively, for 30%, 40%, 50%, 60%, and 70% diameter stenosis, for an increase in aortic pressure from 60 to 140 mm Hg.

**Conclusions:**

The fractional flow reserve value, an index of physiological stenosis significance, was observed to decrease with increasing aortic pressure for a given stenosis in this idealized in vitro experiment for vascular groups with and without myocardial infarction.

## Introduction

Management of stable coronary artery disease requires both anatomical and functional evaluation.^1^, ^2^ Historically, anatomical assessment through invasive coronary angiography was the reference standard to indicate the presence, location, and extent of a stenosis; however, the relationship between the angiographic obstruction and the ischemic potential of a stenosis is complex and cannot be accurately determined by angiography alone. Consequently, functional evaluation of coronary physiology using intracoronary pressure and flow measurements have emerged as important adjunctive measures to determine the ischemic significance of a stenotic lesion and to help guide the clinical decision‐making process. With recent technological advances in sensor‐tipped guidewires, functional parameters are increasingly being used to assess coronary lesion severity at the time of invasive coronary angiography. Pressure‐based fractional flow reserve (FFR; the ratio of average distal pressure [P_d_] and proximal pressure [P_a_] to a stenosis at maximal hyperemia), owing to its extensive clinical outcome validation, has been established as the current gold standard to determine whether a coronary artery lesion is flow limiting and thus potentially responsible for the occurrence of ischemia following increases in myocardial oxygen demands.^3^, ^4^, ^5^

FFR, originally conceptualized based on flow, is defined as the ratio of maximal hyperemic flow through a stenotic artery to that in a hypothetical case in which the artery was normal. Assuming that the venous pressure is negligible and the microcirculatory resistance is minimal and equal in both the normal and stenosed arteries (during hyperemia), the flow‐based ratio can be approximated to a pressure‐based ratio.^6^, ^7^, ^8^, ^9^ FFR can thus be measured as the ratio of average pressures distal (P_d_) and proximal (P_a_) to a stenosis, at maximal hyperemia, induced by a hyperemic agent. Theoretically, in the absence of collaterals, FFR has a lower bound of 0, representing complete vessel obstruction, and an upper bound of 1, representing no obstruction and normal flow. Previous clinical outcome studies have established an FFR cutoff value for deciding whether to revascularize a lesion immediately (FFR ≤0.80) or to defer intervention (FFR >0.80). This cutoff includes a “gray zone” (FFR 0.75–0.8)^10^ in which lesions with FFR ≤0.75 are associated with inducible myocardial ischemia with accuracy >90%.^6^, ^7^, ^11^, ^12^ If the FFR value of a stenosed artery falls within the gray zone, it is suggested that the decision to revascularize should be based on clinical judgment.

Achieving maximal hyperemia is a critical prerequisite to correctly assess FFR. Failure to achieve maximal hyperemia (elevated flow) may result in underestimation of pressure drop across a stenosis, overestimation of FFR, and thus leading to misdiagnosis of ischemia‐causing lesions. Moreover, the choice of hyperemic agent can have a differential effect on hemodynamics.^13^ In addition, in the presence of microvascular dysfunction, the maximal hyperemic flow may not be fully achieved and thus may indicate falsely higher FFR values, thus underestimating the severity of stenosis.

Although FFR is arguably considered to be independent of hemodynamic conditions like blood pressure and heart rate,^9^, ^12^, ^14^, ^15^, ^16^ previous theoretical models have suggested that pressure‐based FFR is affected by changes in hemodynamics, especially absolute aortic pressure and hyperemic flow rate.^17^ Consequently, the objective of this study was to use an in vitro experimental model to characterize coronary circulation in idealized models and to assess the effect of variation of flow rate and aortic pressure (P_a_ ranging from hypotension [60 mm Hg] to hypertension [140 mm Hg]) on the pressure ratio (P_d_/P_a_). The in vitro experimental data were also coupled with hyperemic pressure–flow relationships from human clinical data representing vascular conditions for both myocardial infarction (MI) and non‐MI populations to evaluate the hyperemic pressure ratio (FFR) and to estimate the effect of variation in P_a_ on FFR.

## Methods

An in vitro flow circulation system representative of invasive measurements in a cardiac catheterization laboratory was used to experimentally evaluate the effects of a range of physiological flow rates and aortic pressures on the pressure ratio for different levels of stenosis. The details of the experimental setup are discussed below.

### Stenotic Models

**Figure 1:**
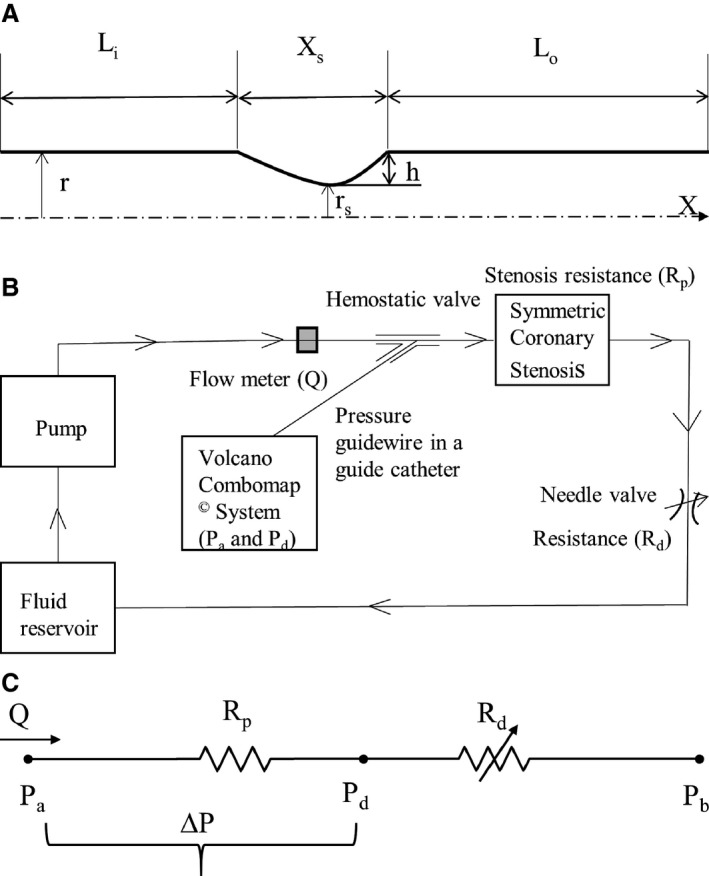
(A) Schematic representation of the idealized axisymmetric stenotic geometry. Nominal vessel diameter D = 2r = 4 mm. (B) Schematic respresentation of the idealized in vitro coronary flow‐loop setup. (C) Electrical analog model of the flow loop with 2 resistances in series. L_i_ indicates inlet length; L_o_, outlet length; r, radius of native vessel; r_s_, minimum radius at the site of stenosis; h, maximum height of stenosis at the throat; x_s_, stenosis length. ΔP, pressure drop across stenosis; P_a_, aortic pressure; P_b_, coronary outflow pressure; P_d_, pressure distal to the stenosis; Q, flow through stenosed artery measured by the flowmeter; R_d_, coronary microvascular resistance; R_p_, stenosis resistance.

Five idealized models with 30%, 40%, 50%, 60%, and 70% diameter stenosis (DS) were fabricated using VeroClear rigid material in an Objet260 Connex printer (Stratasys Ltd.). The stenosis sections were modeled as axisymmetric (Figure 1A) with a smooth gaussian profile, as proposed by Ahmed and Giddens.^18^ The shape of the stenosis was defined by a gaussian profile dependent on axial coordinate x:

$$\begin{array}{cc} & \text{r(x)}=r-\frac{h}{2}\left[1+\text{cos}\left(\frac{\pi x}{x_{s}}\right)\right]\;\text{for}\;\left|x\right|\geq x_{s}\\ & \;\text{r(x)}=\text{r for}\;|x|\leq x_{s} \end{array}$$

In this statement, r is the radius of the native vessel, r_s_ is the radius at the throat of stenosis (Figure 1A), h is the maximum height of stenosis at the throat, and x_s_ is the maximum width of stenosis. The native vessel diameter (D) was 4 mm, and the total length of the section was 116 mm. A 6D (∼24 mm) length was provided prior to the stenosis (L_i_) with a 20D (∼80 mm) length after the stenosis (L_o_) so that the flow becomes fully developed when exiting the stenosis model. The geometric dimensions of the 5 stenosis models (30%, 40%, 50%, 60%, and 70% DS) are tabulated in Table 1.

**Table 1:** Reduction of Stenotic Area Against Equivalent Reduction of Nondimensional Ratio

h/r	%DS	%AS	h (mm)	L_i_ (mm)	X_s_ (mm)	L_o_ (mm)	L_total_ (mm)
0.3	30	0.51	0.6	24	12	80	116
0.4	40	0.64	0.8	24	12	80	116
0.5	50	0.75	1	24	12	80	116
0.6	60	0.84	1.2	24	12	80	116
0.7	70	0.91	1.4	24	12	80	116

%AS indicates percentage area stenoses; %DS, percentage diameter stenosis; h, maximum height of stenosis at throat; h/r, nondimensional ratio; L_i_, inlet length before stenosis section; L_o_, outlet length after stenosis section; L_total_, total length of the model (L_i_+X_s_+L_o_); X_s_, length of stenosis section.

### In Vitro Flow Circulation System

The coronary flow system, shown in Figure 1B, was used to perform the in vitro experiments under physiological steady flow conditions of pressure and flow. The flow was maintained to be quasisteady in the flow system, and the mean flow rate was used as the relevant maximum flow rate scale (perceived hyperemia). A 60:40 mixture (by volume) of distilled water and glycerol (Shelley Medical Imaging Technologies) having viscosity (4.5 cP) and density (1.04 g/cm^3^) similar to those of blood was selected for use in the experiment as the newtonian blood analog fluid.

A Cole‐Parmer digital gear‐drive pump (model EW‐74014‐42) was used to impart and vary the flow rates in the flow system. The circulation system was modeled as flow‐dependent stenosis resistance (R_p_) in series with adjustable downstream resistance (R_d_, needle valve; model EW‐06394‐04). The corresponding electrical analog of the model is shown in Figure 1C. The fluid reservoir was open to atmosphere, thus assuming coronary outflow pressure in Figure 1C to be 0. The fluid reservoir, pump, stenosis model, and needle valve were connected to form a closed loop using flexible platinum‐cured silicone tubing. This flexible tube also helped isolate the test section by damping the unwanted structural vibrations, if any, generated at the pump.

### Experimental Setup

To mimic the pressure measurement in a cardiac catheterization laboratory setting, a 5F diagnostic catheter was advanced proximal to the stenosis section through a cannula. The aortic pressure (P_a_) was measured through a fluid‐filled line connected to a Namic disposable transducer (Navilyst Medical) and the coronary guiding catheter. A 0.014‐in pressure sensor–tipped guidewire connected to a Volcano ComboMap machine (Volcano Corporation) was set to zero and advanced via an introducer needle and a hemostatic valve through the diagnostic catheter. The pressure sensor–tipped guidewire was then calibrated, normalized to the diagnostic catheter, and advanced distal to the stenosis section. The pressure distal to the stenosis (P_d_) was measured through this pressure sensor–tipped guidewire. Inlet flow rate into the stenosis test section was measured using a transit‐time ultrasound clamp‐on flow sensor (TS410‐ME4PXL; Transonic Inc).

### Experimental Protocol

**Figure 2:**
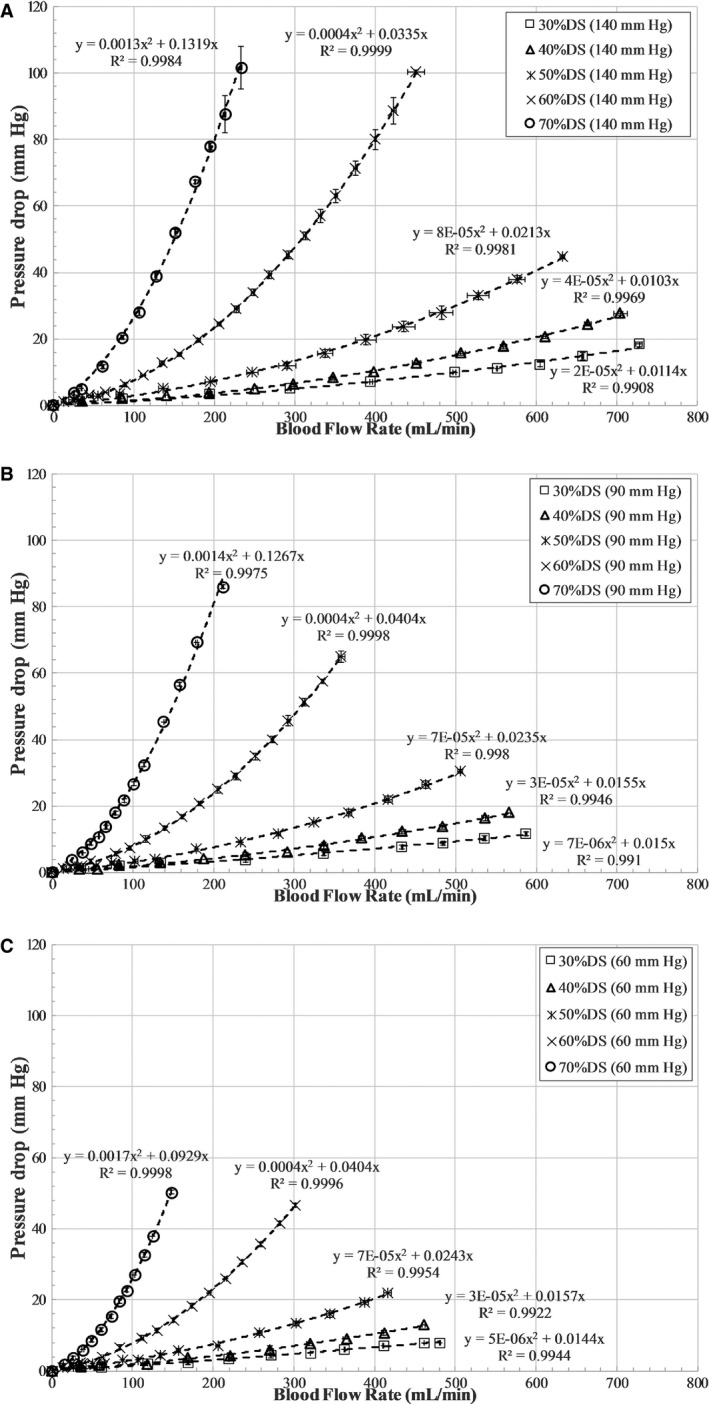
Pressure drop (ΔP=P_a_−P_d_) vs flow characteristics for all stenosis models at fixed aortic pressures of (A) 140 mm Hg, (B) 90 mm Hg, and (C) 60 mm Hg. DS indicates diameter stenosis; P_a_, aortic pressure in mm Hg; P_d_, distal pressure in mm Hg; Q, flow rate in mL/min.

**Figure 3:**
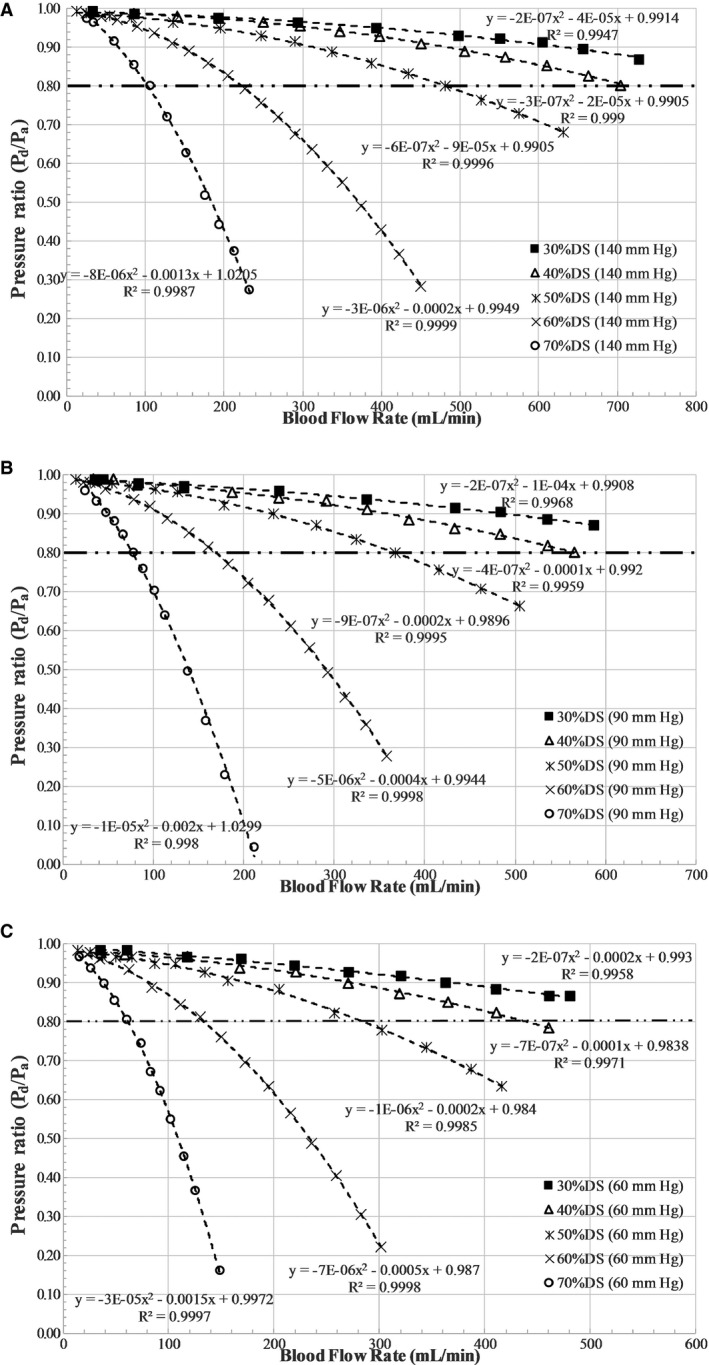
Pressure ratio vs flow characteristics for all stenosis models at fixed aortic pressures of (A) 140 mm Hg, (B) 90 mm Hg, and (C) 60 mm Hg. DS indicates diameter stenosis; P_a_, aortic pressure in mm Hg; P_d_, distal pressure in mm Hg; Q, flow rate in mL/min.

The stenotic models were fixed in the flow system one at a time, as shown in Figure 1B. The blood analog fluid was then allowed to circulate through the flow system for about 5 minutes prior to the experiment to achieve steady‐state conditions, and care was taken so that the flow loop did not have any air bubbles during the experiment. Mean P_a_ was maintained as constant at 7 different levels (140, 120, 100, 90, 80, 70, and 60 mm Hg) to represent conditions of hypertension, normotension, and hypotension during the experiment, reflecting the range of experimental and clinical studies.^8^, ^14^, ^15^, ^17^ The pressure ranges in this study were considered equivalent to the mean or average pressures during the whole cardiac cycle, as measured in conventional FFR. For each stenosis model, under constant P_a_, the pump flow rate was varied between ≈180 and 900 mL/min, and the corresponding inlet flow rates (Q) were measured (using a Transonic clamp‐on flow sensor placed proximally to the stenosis model), along with P_d_ (using the pressure sensor guidewire placed distally to the stenosis). The P_d_ for each varying flow rate was measured only after pulling back the pressure guidewire into the diagnostic catheter and renormalizing and advancing across the stenosis to avoid the effect of drift on the measurements. Mean P_a_ was maintained at a constant level, under different flow rates, by varying the needle valve resistance that mimicked adjustable microcirculatory resistance. The pressure ratio (P_d_/P_a_) at differing prescribed flow rates, applied to each stenosis, was then assessed for all 7 levels of P_a_. Three sets (n=3) of experiments were carried out, and the 3 pressure–flow data sets were averaged to obtain the curve for pressure drop–flow rate (ΔP–Q) (Figure 2) and pressure ratio–flow rate (P_d_/P_a_–Q) (Figure 3) for each of the 5 stenosis test sections at the 7 levels of P_a_.

### Pressure Drop–Flow Rate Characteristics

Young et al^19^, ^20^, ^21^, ^22^, ^23^ developed fluid dynamic equations that describe the relationship between the pressure distal to a stenosis and the flow. The pressure drop–flow rate characteristics across the stenosis can be expressed as a quadratic relationship: ΔP=P_a_−P_d_=A_v_Q+BQ^2^. A_v_ and B are the coefficients for viscous loss along the stenosis and exit loss (due to the change in momentum), respectively. When coronary flow increases, the coronary perfusion pressure distal to the stenosis decreases in a nonlinear fashion, according to the following equation: P_d_=P_a_−A_v_Q−BQ^2^.

### Determination of Hyperemic Flow

**Figure 4:**
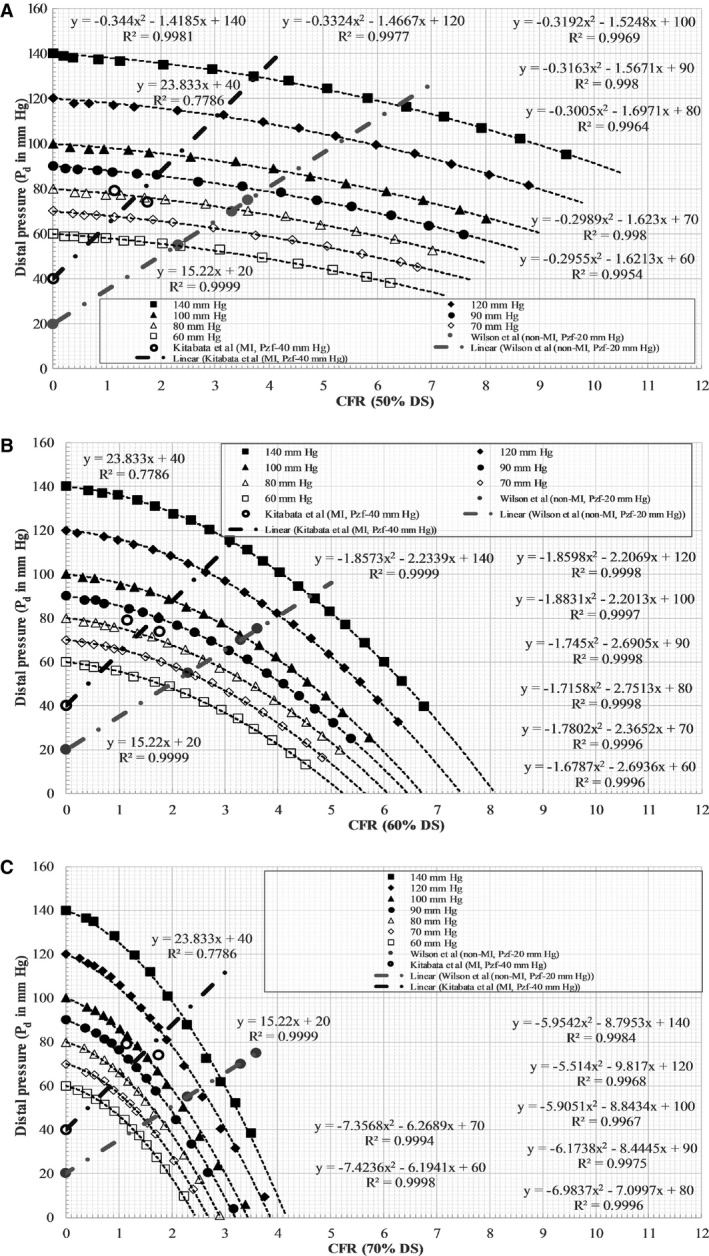
P_d_ vs CFR characteristics for all aortic pressure levels at fixed stenosis of (A) 50% DS, (B) 60% DS, and (C) 70% DS. CFR indicates coronary flow reserve; P_d_, pressure distal to stenosis; DS, diameter stenosis; MI, myocardial infarction; P_zf_, zero‐flow mean pressure; Wilson et al;^26^ Kitabata et al.^27^

The physiological flow conditions, such as pharmacologically induced hyperemia, are unknown in the in vitro experimental setup; however, the methodology for estimating hyperemia using a maximal vasodilation–distal perfusion pressure plot (coronary flow reserve [CFR]–P_d_) was previously proposed by Kirkeeide et al^24^ and reported in an in vitro setting by Roy et al,^25^ assuming a resting blood flow rate of 50 mL/min for a 3‐mm native diameter vessel. Assuming the same Reynolds number flow for both the 3‐ and 4‐mm‐diameter vessels, a resting blood flow rate of 66.67 mL/min was estimated for the 4‐mm native diameter vessel. Utilizing this resting blood flow value, the hyperemic flow rates were obtained using the intersection of the CFR–P_d_ line and the experimental ΔP–Q curve (Figure 4). The CFR–P_d_ line was a linear curve fit based on previously reported clinical data from 2 patient groups with different vascular conditions; a 32‐patient group^26^ with normal microvasculature (non‐MI) and a 27‐patient group^27^ with abnormal microvasculature (MI). These previous clinical studies also reported that the relevant review boards approved their research protocols, and informed consent was obtained from all patients. The non‐MI group had patients with no evidence of MI, no left ventricular hypertrophy, no valvular heart disease, and normal left ventricular ejection fraction; however, the MI patient group consisted of patients who underwent primary percutaneous coronary intervention for a first acute MI (<12 hours from onset of symptoms) and had no valvular disease, no congestive heart failure, and no left ventricular hypertrophy. The *y*‐intercept of the CFR–P_d_ line is denoted as zero‐flow mean pressure (P_zf_), which represents the residual pressure at no flow. Physiologically realistic zero‐flow mean pressure values of 20 mm Hg (non‐MI) and 40 mm Hg (MI), as reported in a previous clinical study,^27^, ^28^ were also used in the maximal vasodilation CFR–P_d_ line (Figure 4). The distal bed resistance offered by the microvasculature to the flow could then be evaluated as follows:

(1) $$R_{d}=\frac{(P_{d}-P_{\mathrm{zf}})}{Q_{h}}$$

### Statistical Analysis

Regression analysis was used to fit a quadratic relationship in (1) pressure drop–flow rate and (2) pressure ratio–flow rate characteristics from the in vitro experiment, respectively, as shown in Figures 2 and 3. Similarly, as demonstrated in Figure 4, linear regression analysis was used to fit a linear curve on the clinical hyperemic CFR–P_d_ characteristics from both non‐MI and MI patients. *R* ^*2*^ values were used to summarize the percentage of variance explained by the regression fit (Figures 2, 3 through 4).

## Results

The pressure drop–flow rate characteristic curves obtained from the in vitro experiment are summarized first, followed by a pressure ratio–flow rate characteristic curve and assessment of FFR with variation in aortic pressure.

### Pressure Drop–Flow Rate Characteristic Curve

Table 2 lists the average values of the coefficient of viscous pressure loss along the stenosis and the coefficient of inertial pressure loss at exit of the stenosis for all 7 P_a_ levels for a given percentage DS. In Table 2, with decreasing stenosis severity, an order of magnitude decrease can be seen in the exit loss coefficient and a relatively small decrease can be seen in the viscous coefficient. These variations indicate that exit loss coefficients are more dominant (higher value) in severely stenosed (70% DS) conditions, whereas viscous loss coefficients are more dominant (higher value) in mildly stenosed conditions (30% DS).

**Table 2:** Viscous and Exit Pressure Loss Coefficients for All Stenosis Models

%DS	D_min_, mm	A_v_, mm Hg/(mL/min)	B, mm Hg/(mL/min)^2^
30	2.8	0.0129	1.17×10^−5^
40	2.4	0.01406	3.3×10^−5^
50	2	0.0234	7.14×10^−5^
60	1.6	0.036743	4×10^−4^
70	1.2	0.118857	1.46×10^−3^

The following equation was used to calculate the coefficients: ΔP=A_v_Q+BQ^2^. %DS indicates percentage diameter of stenosis; A_v_, coefficient of viscous pressure loss along the stenosis; B, coefficient of inertial pressure loss at exit of stenosis; D_min_, minimum diameter at site of stenosis; Q, flow rate in mL/min; ΔP, pressure drop in mm Hg.

Although the experiments were performed over 7 levels of P_a_, the results corresponding to 3 clinically relevant scenarios of hypertension (P_a_=140 mm Hg), normotension (P_a_=90 mm Hg), and hypotension (P_a_=60 mm Hg) are discussed for brevity. The pressure drop–flow rate characteristics for all stenosis models were curvilinear at aortic pressures of 140, 90, and 60 mm Hg, as shown in Figure 2. At P_a_ of 140 mm Hg, the highest flow achieved for 30% DS was 728 mL/min and decreased with increasing severity to 233 mL/min for 70% DS. Similarly, at aortic pressures of 90 and 60 mm Hg, the highest flow rates achieved for 30% DS were 587 and 480 mL/min, respectively, and decreased with increasing stenosis severity to 211 and 148 mL/min, respectively, for 70% DS.

### Pressure Ratio–Flow Rate Characteristic Curve

Figure 3 shows the pressure ratio–flow rate characteristics for all stenosis models. The pressure ratio–flow rate characteristics, similar to pressure drop–flow rate characteristics, demonstrated a curvilinear relationship but with a decreasing slope. Furthermore, the average slope of P_d_/P_a_–Q decreased with increasing DS at all levels of the P_a_. The slope ([mL/min]^−1^) of the P_d_/P_a_–Q curve at the 0.80 ischemic threshold was calculated from the tangent to the curve at a P_d_/P_a_ value of 0.80. The slope for the 30% DS model at the aortic pressure levels of 140, 90 and 60 mm Hg were −3.8216×10^−4^, −3.827×10^−4^, and −4.189×10^−4^, respectively. Similarly, the corresponding slopes for the 70% DS model were −3.0084×10^−3^, −4.0093×10^−3^, and −4.94×10^−3^. When compared among P_a_ levels of 140 and 90 mm Hg, the slope of the P_d_/P_a_–Q curve at the 0.80 ischemic threshold decreased by 0.14% and 33% for 30% and 70% DS, respectively. Similarly, among P_a_ levels of 90 and 60 mm Hg, slope of the P_d_/P_a_–Q curve at the 0.80 ischemic threshold decreased by 9% and 23% for 30% and 70% DS, respectively.

The flow rates corresponding to the intersection of the P_d_/P_a_–Q curve and the P_d_/P_a_ ischemic threshold line (at 0.80) for the 30% DS model at P_a_ of 140, 90 and 60 mm Hg were 942.83, 793.40 and 639.51 mL/min, respectively. Similarly, the flow rates for the 60% DS model at P_a_ of 140, 90, and 60 mm Hg were 222.33, 169.24, and 133.14 mL/min, respectively; flow rates at 70% DS were 102.75, 76.87, and 61.42 mL/min, respectively. Consequently, for 30% DS, the flow rate decreased by 16% ([(942.83–793.40)/942.83]×100) with decreasing P_a_ from 140 to 90 mm Hg and 19% ([(793.40–639.51)/793.40]×100) with decreasing P_a_ from 90 to 60 mm Hg. The corresponding decreases in flow rate were 24% and 21%, respectively, for 60% DS and 25% and 20%, respectively, for 70% DS. It should also be noted that the uncertainty^29^, ^30^ in pressure ratio and flow ratio values due to measurement errors were within 1%.

### Effect of Aortic Pressure on FFR

The hyperemic pressure drop and flow rate were obtained using the intersection of the CFR–P_d_ line and the experimental ΔP–Q curve, as shown in Figure 4 and summarized in Tables 3 and 4. The *y*‐intercept (P_zf_; zero‐flow mean pressure) of the linear fit clinical data line in Figure 4 was 20 mm Hg for the non‐MI patient group and 40 mm Hg for the MI patient group. Such values of zero‐flow mean pressure for non‐MI and MI patients were also reported previously in other clinical studies.^27^, ^28^

**Table 3:** Hyperemic FFR and Flow Rate Estimated From the Coronary Flow Reserve and Distal Pressure characteristics of the Non–Myocardial Infarction Group

P_a_ (mm Hg)	30% DS		40% DS		50% DS		60% DS		70% DS	
	Q_h_ (mL/min)	FFR	Q_h_ (mL/min)	FFR	Q_h_ (mL/min)	FFR	Q_h_ (mL/min)	FFR	Q_h_ (mL/min)	FFR
140	483.90	0.93	466.65	0.90	424.84	0.84	307.46	0.64	193.65	0.46
120	405.14	0.94	392.33	0.91	360.65	0.85	267.77	0.68	170.38	0.49
100	326.82	0.95	316.55	0.92	293.82	0.87	224.45	0.71	144.63	0.53
90	285.23	0.95	278.26	0.93	259.03	0.88	201.32	0.73	130.53	0.55
80	245.97	0.95	240.31	0.94	223.18	0.89	177.47	0.76	116.03	0.58
70	205.37	0.96	200.53	0.94	188.45	0.90	153.69	0.79	101.85	0.62
60	164.18	0.96	161.20	0.95	152.24	0.91	126.40	0.81	86.04	0.66

DS indicates diameter stenosis; FFR, fractional flow reserve; P_a_, aortic pressure in mm Hg; Q_h_, hyperemic flow in mL/min.

**Table 4:** Hyperemic FFR and Flow Rate Estimated From the Coronary Flow Reserve and Distal Pressure characteristics of the Myocardial Infarction Group

P_a_ (mm Hg)	30% DS		40% DS		50% DS		60% DS		70% DS	
	Q_h_ (mL/min)	FFR	Q_h_ (mL/min)	FFR	Q_h_ (mL/min)	FFR	Q_h_ (mL/min)	FFR	Q_h_ (mL/min)	FFR
140	267.75	0.97	264.33	0.96	251.12	0.93	209.05	0.82	145.99	0.66
120	214.47	0.97	212.08	0.97	202.71	0.94	172.82	0.85	121.94	0.70
100	161.48	0.98	158.98	0.97	153.30	0.95	134.13	0.88	96.94	0.75
90	133.87	0.98	132.73	0.97	128.16	0.95	113.06	0.89	83.34	0.78
80	107.62	0.98	106.71	0.98	102.59	0.96	92.10	0.91	69.74	0.81
70	80.83	0.98	79.93	0.98	77.51	0.97	71.18	0.93	55.25	0.85
60	53.73	0.99	53.40	0.98	51.91	0.98	48.07	0.95	38.82	0.90

DS indicates diameter stenosis; FFR, fractional flow reserve; P_a_, aortic pressure in mm Hg; Q_h_, hyperemic flow in mL/min.

**Figure 5:**
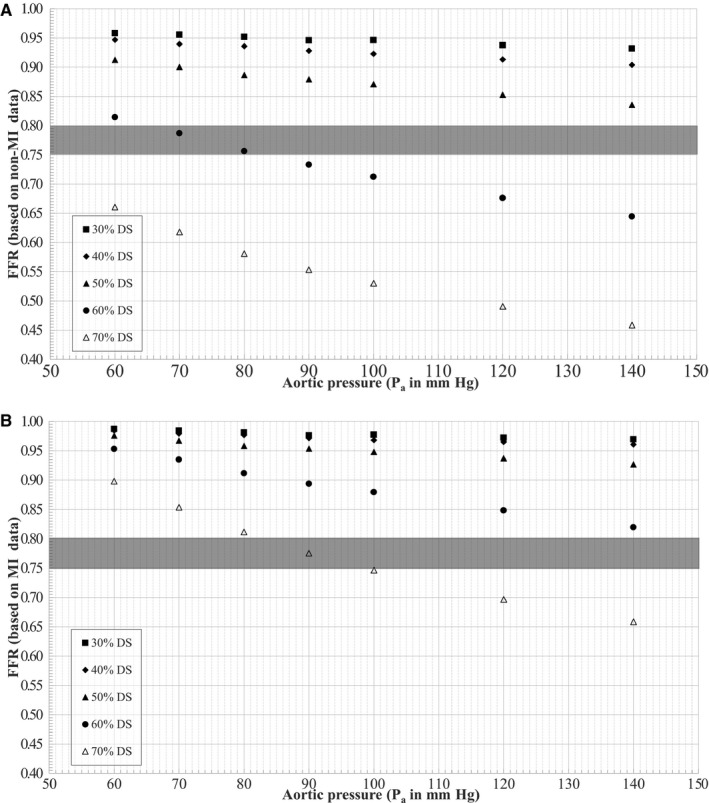
Variation of FFR under (A) non‐MI and (B) MI conditions with aortic pressure. DS indicates diameter stenosis; FFR indicates fractional flow reserve; MI, myocardial infarction; P_a_, aortic pressure.

The variation of FFR, calculated based on intersections of a linear fit of non‐MI patient clinical data^26^ with aortic pressure, in this study is shown in Figure 5A. The microvascular resistance (R_d_) estimated at the hyperemic flow for FFR was 0.23 mm Hg/mL per minute. For a given stenosis, FFR was found to decrease with increasing P_a_ (Figure 5A). The absolute change in FFR was on the order of 0.03, 0.05, 0.07, 0.17, and 0.20 for 30%, 40%, 50%, 60%, and 70% DS, respectively, for an increase in P_a_ from 60 to 140 mm Hg; similarly, the absolute change in FFR was on the order of 0.01, 0.02, 0.03, 0.08, and 0.11, respectively, for an increase in P_a_ from 60 to 90 mm Hg.

In comparison, the variation of FFR, based on MI patient clinical data,^27^ with aortic pressure for the models used in this study is shown in Figure 5B. The microvascular resistance (R_d_) estimated at the hyperemic flow for FFR was 0.36 mm Hg/mL per minute. Similar to the non‐MI group, FFR for a given stenosis with MI was found to decrease with increasing P_a_ (Figure 5B). The absolute change in FFR was slightly higher, on the order of 0.02, 0.02, 0.05, 0.13, and 0.24 for 30%, 40%, 50%, 60%, and 70% DS, respectively, for an increase in P_a_ from 60 to 140 mm Hg; similarly, the absolute change in FFR was on the order of 0.01, 0.01, 0.03, 0.06, and 0.12, respectively, for an increase in P_a_ from 60 to 90 mm Hg. Notably, as shown in Figure 5B, for 70% DS, the FFR values progressively decreased with increasing P_a_ while transitioning through the gray zone, indicating a possible misinterpretation and misdiagnosis of stenoses in main coronary arteries for MI patients.

## Discussion

An in vitro experimental flow loop was developed to model physiological coronary circulation in idealized geometry as flow‐dependent stenosis resistance in series with downstream resistance. Five 3D printed stenosis models of 30%, 40%, 50%, 60%, and 70% DS were used to evaluate the pressure–flow characteristics for 7 levels of aortic pressures ranging from 140 to 60 mm Hg. The pressure drop–flow rate characteristics replicated the standard quadratic form of ΔP=A_v_Q+BQ^2^. The pressure ratio–flow rate characteristics, similar to pressure drop–flow rate, demonstrated a curvilinear relationship but with decreasing slope. More Importantly, when the in vitro experimental data were coupled with hyperemic pressure–flow relationships from human data,^26^, ^27^ it was observed that hyperemic FFR for a given stenosis was influenced by aortic pressure. Such an influence on FFR, especially near the gray zone (FFR 0.75–0.80) can lead to misinterpretation of ischemic severity of a lesion (Figure 5). Consequently, FFR values should be interpreted cautiously in patients with lower mean aortic pressures (hypotension) for clinical decision making during cardiac catheterization.

### Hyperemic Pressure and Flow in MI and Non‐MI Patients

It should be noted that the microcirculation is generally preserved in non‐MI patients. In patients with MI, however, the microcirculation may be severely injured and may compromise the response to hyperemia, leading to reduced hyperemic flow and pressure drop and thus increased pressure‐based FFR. The difference in hyperemic flow rate between MI and non‐MI patients at an aortic pressure of 90 mm Hg was 53%, 52%, 51%, 44%, and 36% for 30%, 40%, 50%, 60%, and 70% DS, respectively (the associated difference in FFR was 3%, 5%, 8%, 44%, and 40%, respectively). With the percentage differences ranging from 3% to 44%, FFR values remained higher in MI patients than in non‐MI patients. More Importantly, it should be noted that the higher difference in FFR values (overestimation) for 70% DS suggests the effect of MI (abnormal microvasculature) that may lead to misdiagnosis of stenosis severity in main coronary arteries by FFR. Furthermore, similar to the above in vitro results, Claeys et al.,^31^ in a clinical study using intracoronary pressure and flow measurements from MI and non‐MI patients with mean lesion severity of 44% DS have also reported that a reduction of maximal flow rate by 23% was associated with 5% increase in FFR.

### Comparison With Previous Studies

Although FFR is a simplified pressure‐based parameter, it is considered to be independent of hemodynamic conditions based on previously reported animal^8^ and human^14^ studies. Nevertheless, it should be noted that in these studies, variation of arterial pressure (hypotension) was achieved using nitroprusside, which induces reflex tachycardia and may have affected the FFR values.^17^ It is noteworthy that Nijjer et al^32^ recently reanalyzed the findings from the animal study^8^ using Bland‐Altman analysis and reported that FFR is indeed altered by pressure changes, in line with results from the present work. Furthermore, Siebes et al^17^ used a theoretical approach with a resistive model of epicardial stenosis and reported that for a given stenosis, FFR increased with decreasing P_a_, similar to the present in vitro findings.

In a preclinical animal study, Gould et al^15^ proposed a parameter, relative flow reserve (ratio of maximal flow with stenosis to normal maximal flow without stenosis; flow‐based formulation of FFR), to more accurately describe stenosis severity. Their results of relative maximal flow for the intermediate stenosis range demonstrated noticeable variability (43% change in relative flow reserve for 61% DS) with changes in aortic pressure from 70 to 150 mm Hg. Consistent with the results in that preclinical study, the present in vitro study showed 21% ([(0.81–0.64)/0.81]×100) variability in the pressure‐based FFR (Table 3) for a 60% DS model with changes in aortic pressure from 60 to 140 mm Hg. Even though the percentage values are different, presumably because of flow‐ and pressure‐based estimation, a similar consistent variability can be observed. It should also be noted that with a considerable fall in P_a_, a paradoxical vasoconstriction of the microcirculatory bed can occur to preserve tissue perfusion.^32^ This is observed as a paradoxical rise in P_d_ in the face of falling P_a_ during intravenous adenosine infusion, leading to an increase in the ratio between the 2 parameters (ie, FFR). In such circumstances, FFR values are considered uninterpretable, and efforts should be made to restore blood pressure before measuring FFR.^33^

In a prospective clinical study, Verdier‐Watts et al^33^ enrolled 12 patients with stable angina (non‐MI) who were referred for invasive cardiac catheterization and were also showing arterial hypotension during FFR measurement. The average stenosis severity in this patient population was at 58±21% DS. In these patients, FFR was first measured under the baseline hypotension and then under the elevated blood pressure (P_a_) achieved using phenylephrine. It was reported that the FFR measured under baseline hypotension (0.81±0.11) was significantly higher than the corresponding FFR under elevated P_a_ after phenylephrine injection (0.75±0.12). In line with these clinical data for non‐MI patients, the FFR values for a similar 60% DS in this in vitro study (Figure 5A) also progressively decreased with increasing P_a_ while transitioning through the gray zone, indicating possible misinterpretation and misdiagnosis.

Tarkin et al^34^ recently performed a large retrospective analysis of catheter data in 283 patients (310 coronary stenoses) and studied the hemodynamic response of intravenous adenosine and its effects on systemic and coronary blood pressure and FFR. In the assessment of intermediate stenosis, the authors reported that P_a_ is, on average, responsible for the majority of the fall in P_d_. Moreover, when there is a large drop in P_a_, the apparent drop in P_d_/P_a_ calculation may not represent worsening stenosis but rather be caused by the lower values of P_a_ and P_d_. If, for example, the pressure drop were preserved but P_a_ and P_d_ were lowered, the pressure ratio would also be lowered. This result gives the false appearance that the stenosis has increased in physiological significance but in fact is related to simple mathematics for ratio calculations^34^ associated with FFR. Similarly, in line with these clinical observations, the results from this in vitro experimental study also demonstrated the variation of FFR (P_d_/P_a_) with arterial pressure. Furthermore, in this clinical study,^34^ it was also reported that intravenous adenosine results in changes in systemic blood pressure and can lead to alterations in FFR lesion classification, potentially affecting clinical management decisions. When FFR measurements were made at peak and stable hyperemic pressures, differences in classification of 9% and 5.2% of cases were observed for FFR treatment thresholds of 0.8 and 0.75, respectively. In addition, peak and stable FFR values from the entire study crossed above and below the 0.8 threshold, suggesting that alteration is an important problem with adenosine assessment.

### Assumptions

The wall of the stenosis geometry was assumed to be rigid in the in vitro experiment. A rigid wall approximation compared with a compliant wall model is expected to provide a conservative estimate^35^ (limiting case) of pressure drop, as seen in hyperemia; however, further in vitro experiments with compliant stenosis models are needed for comparison.

The resting blood flow was assumed to be a constant value of 66.67 mL/min in this study. Previously, in a preclinical study with anesthetized dogs, Gould et al^36^ reported that progressive reduction of coronary lumen has no effect on resting blood flow until the vessel is occluded by ≈80% to 85% of the nominal vessel diameter. More recently, in a large data set of real‐world patients who underwent simultaneous intracoronary pressure and flow measurement, Nijjer et al^37^ also reported that resting flow is preserved despite increasing stenosis severity owing to compensatory reduction in resting microvascular resistance.

### Limitations

Geometric parameters such as shape, length of stenosis, percentage of DS, and symmetry conditions are a few of the parameters expected to influence pressure drop across a stenosis.^38^, ^39^ Further investigation is necessary.

Steady‐state average pressure and flow values in physiologically realistic ranges were used in this in vitro experiment because FFR values are defined as the mean pressure ratios. In an in vitro experiment, Huo et al^40^ previously compared pressure drop between pulsatile flow and steady‐state flow. They reported that pressure drop across a stenosis remained relatively unchanged (<5%) provided that the mean value of the pulsatile flow rate (time averaged over a cardiac cycle) equaled the steady‐state value. Nevertheless, we plan to extend the present work to study the effects of unsteady pulsatile flow. The blood analog fluid used in the in vitro model has a newtonian viscosity of 4.5 cP, similar to normal blood viscosity data available in the existing literature. The viscosity of blood changes with many factors and may affect pressure drop somewhat because of variability in viscous losses.

The experiments were conducted with an idealized single arterial stenosis model, neglecting the effect of bifurcation, serial lesions, or collateral flow, which may cause additional levels of pressure drop. Consequently, future studies using patient‐specific 3D printed models that account for the presence of bifurcation, serial lesions or collateral flow should extend our current work.

## Conclusion

In this work, an in vitro experimental flow loop was developed to model physiological coronary circulation in idealized geometry as flow‐dependent stenosis resistance in series with a downstream resistance. The pressure ratio–flow rate characteristics demonstrated a curvilinear relationship with decreasing slope. In addition, our main finding was that the pressure ratio at maximal hyperemia (FFR) value was observed to decrease with increasing aortic pressure (P_a_ range 60–140 mm Hg) for a given stenosis. More Importantly, for intermediate lesions, the same stenosis (eg, 60% DS in Figure 5A and 70% DS in Figure 5B) can have FFR values above and below the clinical cutoff values (FFR 0.80) at different aortic pressure levels and flow conditions. With the increasing use of FFR in routine clinical practice, it may be worthwhile to understand the significance of its hemodynamic dependency and to interpret the FFR values carefully for clinical decision making based on hemodynamic conditions at the time of measurement.

## Sources of Funding

This manuscript was supported, in part, by grants from the National Institutes of Health, the National Heart, Lung, and Blood Institute (R01 HL118019, R01 HL115150 and R21 HL132277), as well as from a generous gift from the Dalio Foundation.

## Disclosures

Dr Min serves as a consultant to HeartFlow, Inc.; and serves on the medical advisory board of Arineta. He owns equity in MDDX and Autoplaq. None of other authors have any conflicts of interest to declare.
